# Metabolic reprogramming and redox adaptation in sorafenib-resistant leukemia cells: detected by untargeted metabolomics and stable isotope tracing analysis

**DOI:** 10.1186/s40880-019-0362-z

**Published:** 2019-04-04

**Authors:** Xin You, Weiye Jiang, Wenhua Lu, Hui Zhang, Tiantian Yu, Jingyu Tian, Shijun Wen, Guillermo Garcia-Manero, Peng Huang, Yumin Hu

**Affiliations:** 1 Department of Experimental Therapeutics, Sun Yat-sen University Cancer Center, State Key Laboratory of Oncology in South China, Collaborative Innovation Center for Cancer Medicine, 651 Dong Feng East Road, Guangzhou, 510060 Guangdong P. R. China; 2 The First Department of Chemotherapy, The First Affiliated Hospital of Fujian Medical University, Fuzhou, 350005 Fujian P. R. China; 3 Metabolic Innovation Center, Zhongshan School of Medicine, Sun Yat-sen University, Guangzhou, 510080 Guangdong P. R. China; 4 Department of Leukemia, The University of Texas M.D. Anderson Cancer Center, Houston, TX 77030 USA

**Keywords:** FLT3/ITD, Metabolomics, Glycolysis, Antioxidants, Resistance, Sorafenib, Acute myeloid leukemia, Sorafenib, Leukemia

## Abstract

**Background:**

Internal tandem duplications (ITD) within the juxtamembrane domain of FMS-like tyrosine kinase 3 (FLT3) represent a poor prognostic indicator in acute myeloid leukemia (AML). Therapeutic benefits of tyrosine kinase inhibitors, such as sorafenib, are limited due to the emergence of drug resistance. While investigations have been conducted to improve the understanding of the molecular mechanisms underlying the resistance to this FLT3 inhibitor, a profile of cell functioning at the metabolite level and crosstalk between metabolic pathways has yet to be created. This study aimed to elucidate the alteration of metabolomic profile of leukemia cells resistant to the FLT3 inhibitor.

**Methods:**

We established two sorafenib-resistant cell lines carrying FLT3/ITD mutations, namely the murine BaF3/ITD-R and the human MV4-11-R cell lines. We performed a global untargeted metabolomics and stable isotope-labeling mass spectrometry analysis to identify the metabolic alterations relevant to the therapeutic resistance.

**Results:**

The resistant cells displayed fundamentally rewired metabolic profiles, characterized by a higher demand for glucose, accompanied by a reduction in glucose flux into the pentose phosphate pathway (PPP); and by an increase in oxidative stress, accompanied by an enhanced glutathione synthesis. We demonstrated that the highest scoring network of altered metabolites in resistant cells was related to nucleotide degradation. A stable isotope tracing experiment was performed and the results indicated a decrease in the quantity of glucose entering the PPP in resistant cells. Further experiment suggested that the inhibition of major enzymes in the PPP consist of glucose-6-phosphate dehydrogenase deficiency (G6PD) in the oxidative arm and transketolase (TKT) in the non-oxidative arm. In addition, we observed that chronic treatment with sorafenib resulted in an increased oxidative stress in FLT3/ITD-positive leukemia cells, which was accompanied by decreased cell proliferation and an enhanced antioxidant response.

**Conclusions:**

Our data regarding comparative metabolomics characterized a distinct metabolic and redox adaptation that may contribute to sorafenib resistance in FLT3/ITD-mutated leukemia cells.

**Electronic supplementary material:**

The online version of this article (10.1186/s40880-019-0362-z) contains supplementary material, which is available to authorized users.

## Background

Internal-tandem duplications (ITDs) in the FMS-like tyrosine kinase 3 (FLT3) gene are frequently identified genetic alterations in acute myeloid leukemia (AML) [1]. Small-molecule tyrosine kinase inhibitors (TKI) against FLT3/ITD-driven leukemia, including sorafenib, have shown clinical efficacy on AML [2, 3]. However, patient responses tend to be transient due to the emergence of drug resistance [4]. Relapse or disease progression is commonly accompanied by the acquisition of point mutations in the leukemia cells. Non-mutational mechanisms include the activation of the PI3K/AKT and Mitogen-activated protein kinases/Extracellular signal-regulated kinases (MAPK/ERK) pro-survival pathways, among others [5]. While a number of studies have revealed the molecular mechanisms underlying the resistance to FLT3 TKI, a profile of metabolic cell functioning remains to be elucidated.

We have previously shown that the constitutive activation of FLT3/ITD attenuates mitochondrial respiration and promotes glycolytic activity [6]. Such metabolic conversion, termed aerobic glycolysis or the Warburg effect, has been recognized as a “hallmark” of cancer [7]. Although an increasing number of studies on metabolic alterations in cancers have been reported, including leukemia [8], the adaptations of cancer metabolism in the context of therapeutic resistance have not been fully investigated. Our recent study demonstrated that FLT3/ITD-positive leukemia cells resistant to tyrosine kinase inhibitors possess fundamental metabolic alterations characterized by mitochondrial dysfunction and upregulation of glycolysis [9]. It is known that the elevated glycolytic activity provides adenosine triphosphate (ATP) and carbons as energy sources and building blocks for tumor growth. Metabolites are the end products of this ongoing metabolism in various pathways.

To characterize the metabolic interplay between glucose utilization, fluctuation in biosynthesis, and modulation of oxidative stress, we applied a global untargeted metabolomics approach to profile the metabolite differences between sorafenib-sensitive and -resistant leukemia cells with FLT3/ITD mutation. As the detailed metabolic alterations had previously remained unknown, this study aimed to characterize the metabolic and redox adaptation profiles and may contribute to the understanding of sorafenib resistance in FLT3/ITD-mutated leukemic cells.

## Materials and methods

### Cell culture condition and establishment of a sorafenib-resistant cell line, BaF3/ITD-R

The mouse BaF3 hematopoietic progenitor cell line transfected with FLT3/ITD (BaF3/ITD) was kindly provided by Dr. Donald Small (Johns Hopkins University, Baltimore, MD, USA).The human leukemia cell line, MV4-11, expressing the FLT3/ITD mutation was obtained from the American Type Culture Collection (Manassas, VA, USA). Generation of the sorafenib-resistant cell lines (BaF3/ITD-R, MV4-11-R) and the culture conditions used were both performed as previously described [9].

### Global untargeted metabolomics with liquid chromatography–mass spectrometry (LC–MS)

An equal number of BaF3/ITD and BaF3/ITD-R cells (~ 5×10^6^ cells) in the exponential growth phase were collected in 1.5-mL Eppendorf tubes and rinsed twice with 1 mL of ice-cold normal saline solution. Metabolite extraction and analysis with ultra-high-performance liquid chromatography electrospray ionization mass spectrometry (UHPLC-ESI–MS) analysis (Q Exactive; Thermo Fisher Scientific, Inc., Waltham, MA, USA) was performed as follows. One mL of ice-cold MilliQ water (Millipore, Burlington, MA, US) was added and the cell suspensions were lysed by two freeze–thaw cycle (frozen in liquid nitrogen and thawed at 37 °C for 10 min), followed by 30 s of sonication on ice. Next, 900 μL pre-chilled methanol (− 20 °C) was added to 300 μL cell suspension. The mixture was vigorously vortexed and centrifuged at 14,000×*g* for 15 min at 4 °C to precipitate proteins and particulates. The supernatant containing the polar extracts was transferred to a 1.5 mL Eppendorf (Hauppauge, NY, USA) and evaporated overnight. Five biological replicates were prepared for ultrahigh-performance liquid chromatography electrospray ionization mass spectrometry (UHPLC-ESI–MS) analysis (Q Exactive, Thermo Fisher Scientific). Polar metabolites were separated on a HILIC (Hydrophilic interaction chromatography) Silica column (Waters, Milford, MA, USA) with column temperature at 40 °C using a gradient elution program at a flow rate of 300 μL/min. The samples were cooled in an auto-sampler at 10 °C and the injection volume was 5 μL. Samples were run in both positive and negative ionization mode. Mass spectrometric data of polar metabolites was acquired at full scan mode (70–1050 m/z [mass to charge ratio]).

Total ion chromatograms and mass spectra data were generated using the Thermo Scientific SIEVE software (Thermo Fisher Scientific, Waltham, MA, USA). Peak picking, alignment, deisotoping and integration were performed to produce a list of mass and retention time pairs with corresponding intensities for all detected peaks. A two-tailed Student’s t test was used to detect the difference of metabolite intensities between two samples (A *P*-value < 0.05 was considered to be significant). Orthogonal partial least squares-discriminant analysis (OPLS-DA) was used to identify the differential metabolites between two groups using the software SIMCA-P version 14, (Umetrics, Umeå, Sweden). The metabolites were then putatively identified by accurate mass [3 ppm (parts per million) mass error] and fragmentation pattern match (MS/MS spectrum). Structural annotation of the metabolites was searched in public databases including the mz cloud (https://www.mzcloud.org/), HMDB (http://www.hmdb.ca/), and METLIN (http://metlin.scripps.edu).

### Pentose phosphate flux analysis with gas chromatography/mass spectrometry (GC–MS)

Briefly, the cells were washed with 0.9% saline and quenched with 500 µL of − 20 °C methanol. Then 200 µL of ice-cold water containing 1 µg norvaline was added as internal standard, after which 500 µL of − 20 °C chloroform was then added to the samples, vortexed for 15 min and centrifuged at 14,000×*g* for 10 min. The top aqueous layer (polar metabolites) were collected and dried with speed vacuum for GC–MS analysis.

For derivatization, dried polar metabolites were dissolved in 20 µL of 2% (w/v, weight/volume) methoxyamine hydrochloride (Sigma-Aldrich) in pyridine and warmed at 37 °C for 60 min. Subsequent conversion to their tert-butyldimethylsilyl (tBDMS) derivatives was accomplished by adding 30 µL *N*-methyl-*N*-(tert-butyl-dimethylsilyl) trifluoroacetamide and 1% tert-butyldimethylchlorosilane (Regis Technologies) and incubating at 37 °C for 30 min. After centrifugation, the top aqueous layer was collected (~45 µL) and mixed with 55 µL pyridine in 150 µL glass inner tube (Waters).

GC–MS analysis was performed using the Thermo 1300 with a 30-m TG-35MS capillary column (Agilent Technologies) connected to Thermo ISQ QD MS. Electron impact ionization (EI) mode was selected and ionization energy was 70 eV (electronvolt). In splitless mode, 1 µL of the derivatized sample was injected at 270 °C, using helium as the carrier gas at a flow rate of 1.5 mL/min. For analysis of organic and amino acid derivatives, the initial temperature of the GC oven was held at 100 °C for 2 min followed by an increase to 255 °C at a rate of 3.5 °C/min and then ramped to 320 °C at 15 °C/min for a total run time of approximately 50 min. The MS source was held at 300 °C, and the detector was operated in scanning mode, recording ion abundance in the range of 100–650 m/z.

### Determination of cellular NADP^+^/NADPH and GSH levels

Nicotinamide adenine dinucleotide phosphate (NADP^+^) and its reduced form (NADPH) levels were analyzed using an NADP/NADPH Quantification Colorimetric Kit (BioVision, Milpitas, CA, USA), and the total cellular glutathione (GSH) and oxidized glutathione (GSSG) were measured using a Glutathione Fluorometric Assay Kit (BioVision, Milpitas, CA, USA). NADP and NADPH levels were analyzed using NADP/NADPH Quantification Colorimetric Kit (Biovision, Milpitas, CA). A total of 4 × 10^6^ cells were washed with cold PBS and lysed with NADP/NADPH extraction buffer. The supernatant was collected for measurement of NADPH and NADP^+^/NADPH by UV spectrophotometer at 450 nm. Total Cellular glutathione (GSH) and oxidized glutathione (GSSG) was measured using Glutathione Fluorometric Assay Kit (Biovision, Milpitas, CA, USA). Then, 0.4 × 10^6^ cells were prepared by deproteination and subsequent reagents were added following the manufacturer’s instructions. The fluorescence intensity of samples and standards was measured at excitation*/*emission (Ex/Em) wavelengths = 340/420 nm. Cellular GSH and GSSG contents were calculated with the standard curve generated in parallel experiments and normalized to cell counts.

### Quantitative real-time PCR

Total RNA were extracted by Trizol reagent (Invitrogen, Carlsbad, CA, USA). cDNA was synthesized using the PrimerScript RT reagent kit (TaKaRa, Dalian, China). Quantitative real-time PCR was performed using SYBR^®^ Premix Ex Taq™ II (Clontech) and CFX96 real-time PCR detection system (Bio-Rad). Primers used for BaF3/ITD or BaF3/ITD-R cells were GAPDH (F: 5′-TGGATTTGGACGCATTGGTC-3′, R: 5′-TTTGCACTGGTACGTGTTGAT-3′), G6pdx (F: 5′-CACAGTGGACGACATCCGAAA-3′, R: 5′-AGCTACATAGGAATTACGGGCAA-3′), H6pd (F: 5′-AAGATGCTCCTAGCGGCAATG-3′, R: 5′-TCCAGGTATAGCTGAAACAGTCC-3′), Prps1 (F: 5′-ACTTATCCCAGAAAATCGCTGAC-3′, R: 5′-CCACACCCACTTTGAACAATGTA-3′), Prps2 (F: 5′-ATGCCTAACATCGTGCTCTTC-3′, R: 5′-GATCTCGACACTGGTCTCCTG-3′); Rbks(F: 5′-GGTTCCTGCATGACCGACC-3′, R:5′-TGCCAAAAGAATCGTTGCCAA-3′), Rpe (F: 5′-GCACCTGGATGTAATGGACGG-3′, R: 5′-CCTGGCCTAGCTGCTTTCG-3′), Rpia (F: 5′-AAGGCCGAGGAGGCTAAGAA-3′, R: 5′-CTTTCAGCTATTCGCTGCACA-3′), Taldo1 (F: 5′-GTAAAGCGCCAGAGGATGGAG-3′, R: 5′-CTCTTGGTAGGCAGGCATCT-3′); Tkt (F: 5′-ATGGAAGGTTACCATAAGCCAGA-3′, R: 5′-TGCAGCATGATGTGGGGTG-3′). Primers used for MV4-11 or MV4-11-R cells were Actin (F: 5′-CATGTACGTTGCTATCCAGGC-3′, R: 5′-CTCCTTAATGTCACGCACGAT-3′), G6PD (F: 5′-CGAGGCCGTCACCAAGAAC-3′, R: 5′-GTAGTGGTCGATGCGGTAGA-3′), H6PD (F: 5′-GCAGAGCACAAGGATCAGTTC-3′, R: 5′-GGCAGCTACTGTTGATGTTGC-3′), PGLS (F; 5′-GGAGCCTCGTCTCGATGCTA-3′, R: 5′-GAGAGAAGATGCGTCCGGT-3′) PRPS1 (F; 5′- ATCTTCTCCGGTCCTGCTATT-3′, R: 5′-TGGTGACTACTACTGCCTCAAA-3′), PRPS2 (F: 5′-AGCTCGCATCAGGACCTGT-3′, R: 5′- ACGCTTTCACCAATCTCCACG-3′); RBKS (F: 5′-ATGGTCTGCCAGCTCGAAATA-3′, R: 5′-GAGAGGGTGTAGAACTGGGGA-3′),

RPE (F: 5′- CAGGAGCCAATCAGTACACCT-3′, R: 5′-CAAGGCCAACCTGCAATGG-3′), RPIA (F: 5′-AGTGCTGGGAATTGGAAGTGG-3′, R: 5′-GGGAATACAGACGAGGTTCAGA-3′), TALDO1 (F: 5′-CAGCACAGATGCCCGCTTA-3′, R: 5′-CGGCCCGGAATCTTCTTTAGTA-3′); TKT (F: 5′-TCCACACCATGCGCTACAAG-3′, R: 5′-CAAGTCGGAGCTGATCTTCCT-3′).

### Western blot analysis

Collected cells were homogenized in lysis buffer (5% SDS, 10 mM EDTA, 50 mM NaCl, 10 mM Tris–HCl). Protein concentrations were determined using pierce BCA protein assay (Thermo Fisher, Rockford, IL, USA). Proteins were resolved using SDS-polyacrylamide gel electrophoresis and transferred onto nitrocellulose membrane. Membranes were blocked in 5% milk, incubated with primary antibody at a concentration of 1:1000, then incubated with secondary antibody at a concentration of 1:10,000 and read using ECL regent (Bio-Rad, Richmond, CA, USA). Antibodies anti-GCLM, anti-GSS, anti-GCLC, anti-GSR, anti-GPX1, anti-SOD1, anti-SOD2, anti-TKT, anti-G6PD, anti-CGL and anti-Actin were purchased from Abcam. Anti-NRF2 and anti-CBS antibody was purchased from Cell Signaling Technology.

### MTS assay

Cell viability inhibition was determined by MTS assay. Cells were seeded in a 96-well plate with indicated agents for 72 h. Overall, 20 μL MTS reagent (Promega, Madison, WI, USA) was added to each well and incubated for an additional 4 h at 37 °C. The optical density (OD) was determined by a microplate reader (Thermo, Helsinki, Finland) at 490 nm.

### Cell proliferation assay

Cell proliferation was measured by direct cell counting. The cells were seeded in 12-well plates and cultured in the indicated media. The number of cells was counted every 24 h for up to 72 h using a Coulter Z2 Particle Counter & Size analyzer.

### Measurement of intracellular cysteine

Intracellular cysteine levels were measured as previously reported [10]. Cells were harvested and washed with ice-cold PBS, and then sulfosalicylic acid (SSA) was added to each sample. After centrifugation, the supernatant was collected. The pH of supernatant was adjusted to 8.3 using NaOH. Samples were reduced with 5 mM dithiothreitol (DTT) for 15 min at room temperature. Following reduction, samples were acidified with glacial acetic and then reacted with acid ninhydrin reagent for 10 min at 100 °C. The samples were cooled and diluted with 95% ethanol. The absorbance of samples was measured at 560 nm, and cysteine levels were quantified using cysteine hydrochloride standards (0–500 µM) processed in the same manner as the samples.

### Ingenuity pathway analysis (IPA) of differential metabolites

PubChem compound identification number (CIDs) and corresponding fold changes (≥ 1.2 or ≤ 0.80) of differentially involved metabolites between BaF3/ITD and BaF3/ITD-R cells were uploaded to the IPA platform (http://www.ingenuity.com; QIAGEN, Redwood City, CA, USA) in order to identify the relevant biological functions that were most significant in the uploaded datasets.

### Analysis of extracellular fluxes

Extracellular fluxes, including in glucose/glutamine uptake and lactate/glutamate secretion, were measured using a Yellow Springs Instrument 2950D-1 (YSI) Biochemistry Analyzer (YSI Inc, Yellow Springs, Ohio, USA). The quantities (pmol/cell/h) were determined by the difference in substrate concentrations between the final spent medium and the initial medium (RPMI 1640 medium with 10% FBS, GIBCO, Waltham, MA, USA), and normalized by the cell number over 24 h [11].

### Pentose phosphate flux analysis with gas chromatography/mass spectrometry (GC–MS)

A total of 1 × 10^6^ BaF3/ITD or BaF3/ITD-R cells were cultured in a glucose-free RPMI-1640 medium (Gibco, Waltham, MA, USA), supplemented with 11.1 mmol/L [1,2-^13^C_2_]-glucose (Cambridge Isotope Laboratories, Tewksbury, MA, USA) and 10% fetal bovine serum (FBS) in 6-well plates for 24 h. Three biological replicates were prepared. Intracellular metabolites were extracted using a methanol/water/chloroform method as previously described [12]. The extraction, derivatization, and GC–MS analysis procedures were performed as described in above “Pentose phosphate flux analysis with gas chromatography/mass spectrometry” section. Glucose flux through the PPP was calculated as the extracellular glucose uptake flux multiplied by the ratio of (M + 1)/[(M + 1) + (M + 2)], where (M + 1) and (M + 2) are two isotopologues of pyruvate derived from [1,2-^13^C_2_]-glucose.

### Apoptosis and cell cycle assays

The collected cells for apoptosis were stained with Annexin V-FITC and propidium iodide (KeyGEN, Nanjing, China) in binding buffer according to the company’s manual instruction. Apoptosis was determined using FACS Calibur flow cytometer (BD Biosciences, San Diego, CA, USA). For cell cycle analysis, cells were serum starved overnight and stimulated with complete medium for 6 h. Cells were fixed in 70% ethanol, stained with propidium iodide and measured by flow cytometer.

### Statistical analysis

The bars indicate the mean ± standard error of the mean (S.E.M.). The differences between two groups of data were evaluated using the Student’s t test by Prism GraphPad (San Diego, CA, USA). A *P* value < 0.05 was considered as statistically significant.

## Results

### BaF/ITD-R and MV4-11-R cells are highly resistant to sorafenib-induced apoptosis and growth inhibition

**Fig. 1:**
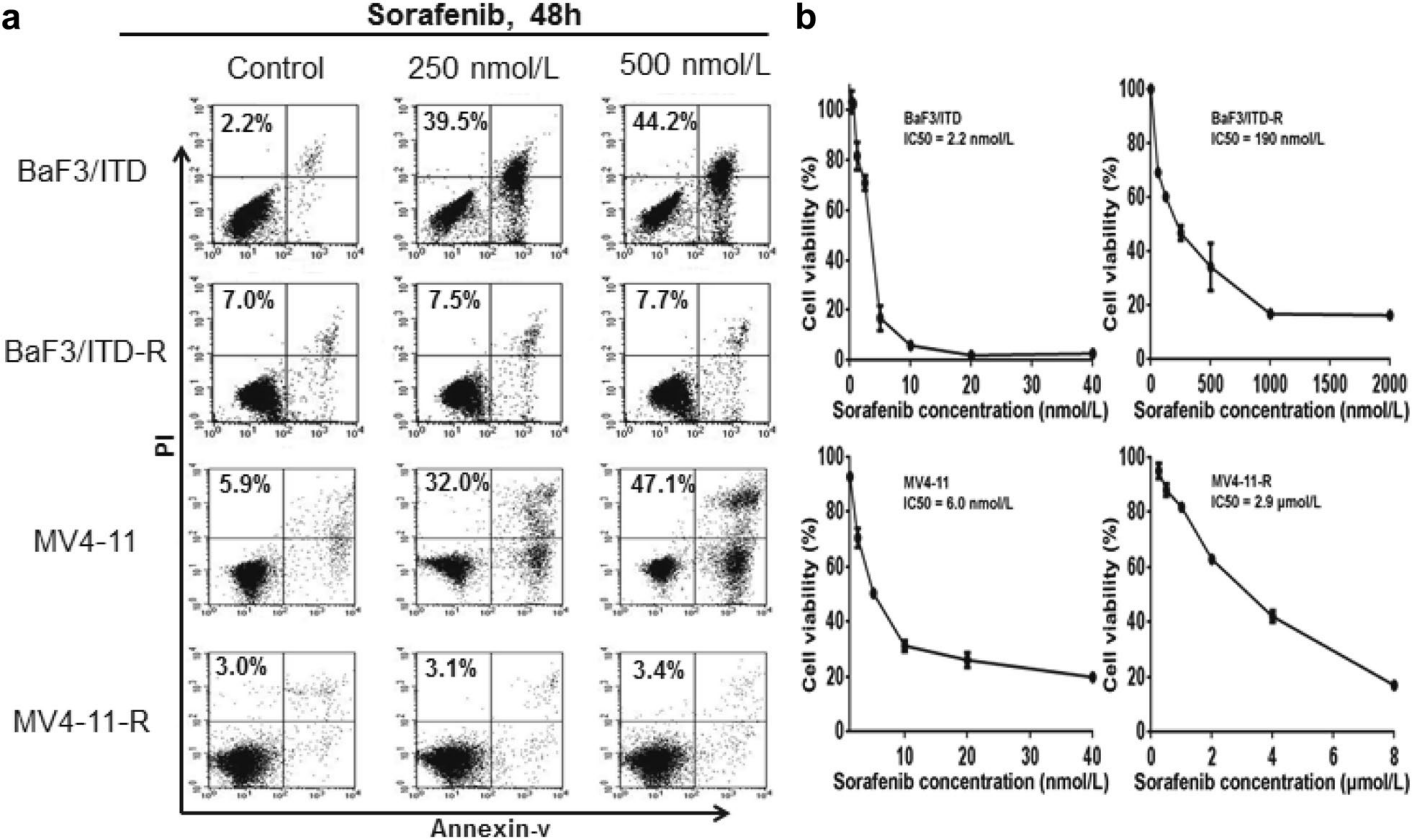
BaF3/ITD-R and MV4-11-R cells are highly resistant to sorafenib. **a** BaF3/ITD, BaF3/ITD-R, MV4-11 and MV4-11-R cells were treated with the indicated concentrations of sorafenib for 48 h and subjected to an Annexin-V/PI assay. The percentage values indicate the total cell death populations. **b** BaF3/ITD, BaF3/ITD-R, MV4-11 and MV4-11-R cells were treated with various concentrations of sorafenib for 72 h and subjected to an MTS assay. The numbers on the curves indicate the IC_50_ of sorafenib in each cell line. *PI* propidium iodide

We established the BaF3/ITD-R and MV4-11-R cell lines as previously described [9]. To validate the resistance of these cell lines, we first compared the cytotoxic effect of sorafenib in the resistant cells (BaF3/ITD-R and MV4-11-R cells) with its effect in the sensitive cells (BaF3/ITD and MV4-11 cells). The cells were treated with 250 and 500 nmol/L sorafenib for 48 h and were then subjected to an Annexin-V/PI assay. As shown in Fig. 1a, at a concentration of 250 and 500 nmol/L, sorafenib induced apoptotic rates of more than 30% and 40% among the populations of the sensitive cells. By contrast, the same concentrations of sorafenib did not cause detectable cell death in the resistant cells. We further compared the inhibitory effect of sorafenib on cell growth using a 72-h 3-(4,5-Dimethylthiazol-2-yl)-5-(3-carboxymethoxyphenyl)-2-(4-sulfophenyl)-2H-tetrazolium, inner salt (MTS) assay. Compared with the parental BaF3/ITD and MV4-11 cells, the BaF3/ITD-R and MV4-11-R cells exhibited markedly higher half maximal inhibitory concentration (IC_50_) values in response to sorafenib (Fig. 1b).

### Metabolomic profiles segregate BaF3/ITD-R cells from BaF3/ITD cells

**Fig. 2:**
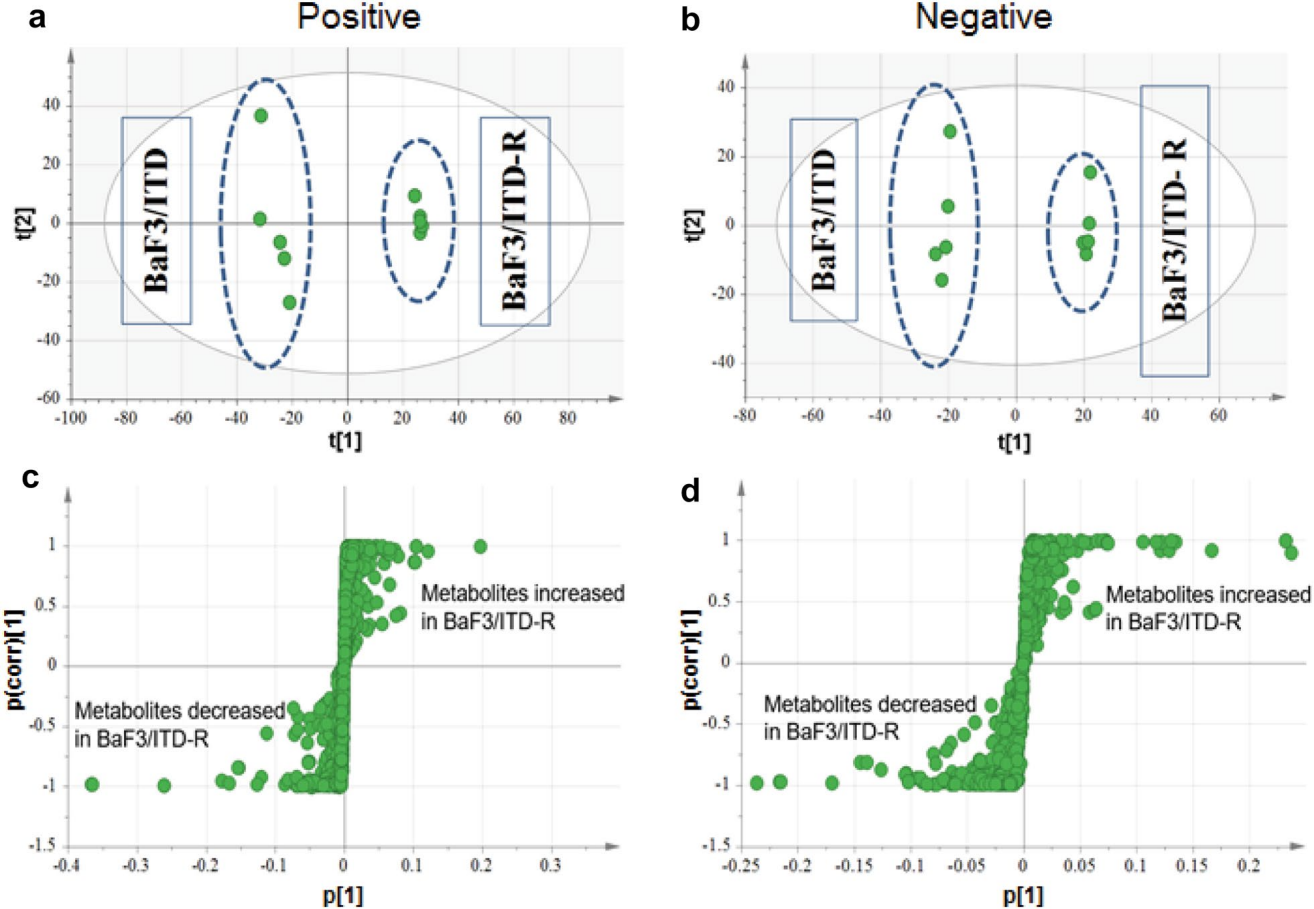
Scores scatter plot and loading S-plot from the supervised OPLS discriminant analyses distinguish the unique metabolomic profiles of BaF3/ITD-R from BaF3/ITD cells. OPLS-DA score plots under the **a** ESI^+^-HILIC mode and **b** ESI^−^-RPLC mode. Highly altered metabolites identified by the S-plot under ESI^+^-HILIC mode (**c**) and ESI^−^-RPLC mode (**d**). Dots in the upper right: metabolites increased in BaF3/ITD-R cells. Dots in the lower left: metabolites decreased in BaF3/ITD-R cells t1 and t2, variables after dimensional reduction; p1, the influence of different metabolites; p corr, the confidence of different metabolites. P corr > 0.5, significant markers; P corr > 0.8, markers with high confidence. *OPLS* orthogonal partial least square, *ESI* electrospray ionization, *HILIC* Hydrophilic interaction chromatography, *RPLC* reversed-phase liquid chromatography

BaF3/ITD and BaF3/ITD-R cells were subjected to global metabolomic analysis. The orthogonal partial least square discriminant analysis (OPLS-DA) indicated a clear distinction in metabolomics between the BaF3/ITD and BaF3/ITD-R cells, suggesting unique metabolomic profiles for sorafenib-sensitive and -resistant cell lines (metabolites in positive mode, R^2^X = 0.666, R^2^Y = 1, Q^2^ = 0.988; metabolites in negative mode, R^2^X = 0.676, R^2^Y = 1, Q^2^ = 0.99) (Fig. 2a, b). The S-plots provide visualization of the OPLS-DA models were used to identify the metabolites that were differentially expressed between the two cell lines (*P* < 0.05, t-test; ITD-R/ITD ratio ≥ 1.2 or ≤ 0.8) (Fig. 2c, d). We observed that there were 40 metabolites with differences in mean intensity at *P* < 0.05 (Table 1). A total of 23 metabolites exhibited higher abundance (ITD-R/ITD > 1.0) and 17 metabolites exhibited lower abundance (ITD-R/ITD < 1.0) in BaF3/ITD-R cells, compared with in BaF3/ITD cells (Table 1).

**Table 1:** Differentially expressed metabolites between BaF3/ITD and BaF3/ITD-R cells

Metabolites name	Retention time	Compound MW	Compound formula	Ratio ITD-R/ITD	*P* value
Guanine	4.38	151.04938	C_5_H_5_N_5_O	9.18	0.0219
Creatine	10.42	131.06949	C_4_H_9_N_3_O_2_	5.13	0.0094
Creatinine	6.78	113.05898	C_4_H_7_N_3_O	3.60	0.0077
l-2-Aminoadipic acid	8.09	161.06883	C_6_H_11_NO_4_	3.19	0.0111
Glyceraldehyde	2.17	90.03173	C_3_H_6_O_3_	2.81	0.0308
5-Hydroxytryptophol	11.13	177.0789	C_10_H_11_NO_2_	2.32	0.0133
l-Glutamate	10.55	147.05315	C_5_H_9_NO_4_	2.29	0.0022
d-Alanine	9.98	89.04769	C_3_H_7_NO_2_	1.91	0.0063
N1-Acetylspermidine	14.57	187.16841	C_9_H_21_N_3_O	1.82	0.0038
Glyceryl phosphate	11.22	172.01370	C_3_H_9_O_6_P	1.73	0.0053
4-Hydroxy-4(3-pyridyl)-butanoic acid	3.39	181.07387	C_9_H_11_NO_3_	1.62	0.0004
4-(3-Pyridyl)-butanoic acid	7.57	165.07907	C_9_H_11_NO_2_	1.61	0.0099
Guanosine	4.49	283.09139	C_10_H_13_N_5_O_5_	1.54	0.0012
l-1-Pyrroline-3-hydroxy-5-carboxylate	8.99	129.04265	C_5_H_7_NO_3_	1.53	0.0027
d-Lysine	13.39	146.10550	C_6_H_14_N_2_O_2_	1.53	0.0125
l-Isoleucine	8.26	131.09465	C_6_H_13_NO_2_	1.48	0.0066
l-Pipecolic acid	13.39	129.07909	C_6_H_11_NO_2_	1.44	0.0100
l-Histidine	8.99	155.06957	C_6_H_9_N_3_O_2_	1.43	0.0014
l-Glutamine	10.04	146.06911	C_5_H_10_N_2_O_3_	1.33	0.0041
Citrulline	10.73	175.09569	C_6_H_13_N_3_O_3_	1.32	0.0017
l-Ornithine	12.73	132.08993	C_5_H_12_N_2_O_2_	1.32	0.0019
l-Methionine	8.17	149.05103	C_5_H_11_NO_2_S	1.30	0.0076
l-Tryptophan	7.36	204.08989	C_11_H_12_N_2_O_2_	1.24	0.0208
Serotonin	6.85	176.09497	C_10_H_12_N_2_O	0.02	0.0062
l-Fucose1-phosphate	10.25	244.03486	C_6_H_13_O_8_P	0.09	0.0035
*S*-Formylglutathione	14.48	335.08000	C_11_H_17_N_3_O_7_S	0.15	0.0029
Uric acid	3.15	168.02832	C_5_H_4_N_4_O_3_	0.18	0.0037
Hypoxanthine	3.15	136.03855	C_5_H_4_N_4_O	0.20	0.0018
Inosine	3.48	268.08068	C_10_H_12_N_4_O_5_	0.22	0.0104
*N*-Acetyl-l-lysine	11.34	188.11614	C_8_H_16_N_2_O_3_	0.36	0.0008
l-Asparagine	9.96	132.05348	C_4_H_8_N_2_O_3_	0.45	0.0017
Trans-4-Hydroxy-l-proline	9.79	131.05832	C_5_H_9_NO_3_	0.42	0.0101
Adenosine	4.42	267.09556	C_10_H_13_N_5_O4	0.52	0.0063
*S*-Adenosylhomocysteine	10.24	384.12145	C_14_H_20_N_6_O_5_S	0.56	0.0005
Glycine	9.34	75.03206	C_2_H_5_NO_2_	0.56	0.0091
Adenine	4.32	135.05455	C_5_H_5_N_5_	0.60	0.0015
l-Carnitine	12.08	161.10523	C_7_H_15_NO_3_	0.61	0.0016
(R)-2-Amino-3-hydroxypropanoic acid	9.51	105.04264	C_3_H_7_NO_3_	0.65	0.0102
*N*-Acetyl-d-galactosamine	4.26	221.08994	C_8_H_15_NO_6_	0.72	0.0009
3-Ketosphinganine	6.29	299.28224	C_18_H_37_NO_2_	0.79	0.0007

### Ingenuity pathway analysis (IPA) of differential metabolites between BaF3/ITD and BaF3/ITD-R

A total of 40 differentially expressed metabolites (Table 1) with PubChem IDs were uploaded to the IPA platform for pathway analysis. Canonical pathways with *P* < 0.05 were defined as significant. The five most significantly different pathways are shown in Table 2. Also, as shown in Table 1, the metabolites with the most pronounced differences in abundance between BaF3/ITD and BaF3/ITD-R cells were guanine (9.18-fold increase in BaF3/ITD-R) and serotonin (50-fold decrease in BaF3/ITD-R). IPA showed that guanine was associated with purine ribonucleoside degradation to ribose-1-phosphate (Additional file 1: Figure S1), which was also the highest ranked canonical pathway in terms of significant changes. Adenine, adenosine, guanosine, hypoxanthine, and inosine were also included in this network with significant changes. As shown in this pathway, guanosine and phosphate are two substrates of the enzyme guanosine phosphorylase, with its two products being guanine and alpha-d-ribose-1-phosphate. Alpha-d-ribose-1-phosphate was then further converted to d-ribose-5-phosphate by phosphopentomutase, after which the d-ribose 5-phosphate, as an intermediate, enters the non-oxidative phase of the PPP, resulting in synthesis of 6-carbon sugars for glycolytic activity [13].

**Table 2:** Ingenuity analysis of top ranked pathways of differentially involved metabolites between BaF3/ITD and BaF3/ITD-R cells

Pathway	Ratio^a^	*P* value
Purine ribonucleoside degradation to ribose-1 phosphate	6/12 (50%)	1.93E−08
Purine nucleotides degradation	6/17 (35.3%)	2.43E−07
Adenine and adenosine salvage	4/10 (40.0%)	1.85E−05
Adenosine nucleotides degradation	4/11 (36.4%)	2.87E−05
Guanosine nucleotides degradation	3/10 (30.0%)	6.35E−04

^a^Ratio: The number of molecules from the focus gene set that map to the pathway to the total number of molecules that map to the canonical pathway

### Rewired glucose flux into the pentose phosphate pathway and glycolytic pathway in BaF3/ITD-R cells

**Fig. 3:**
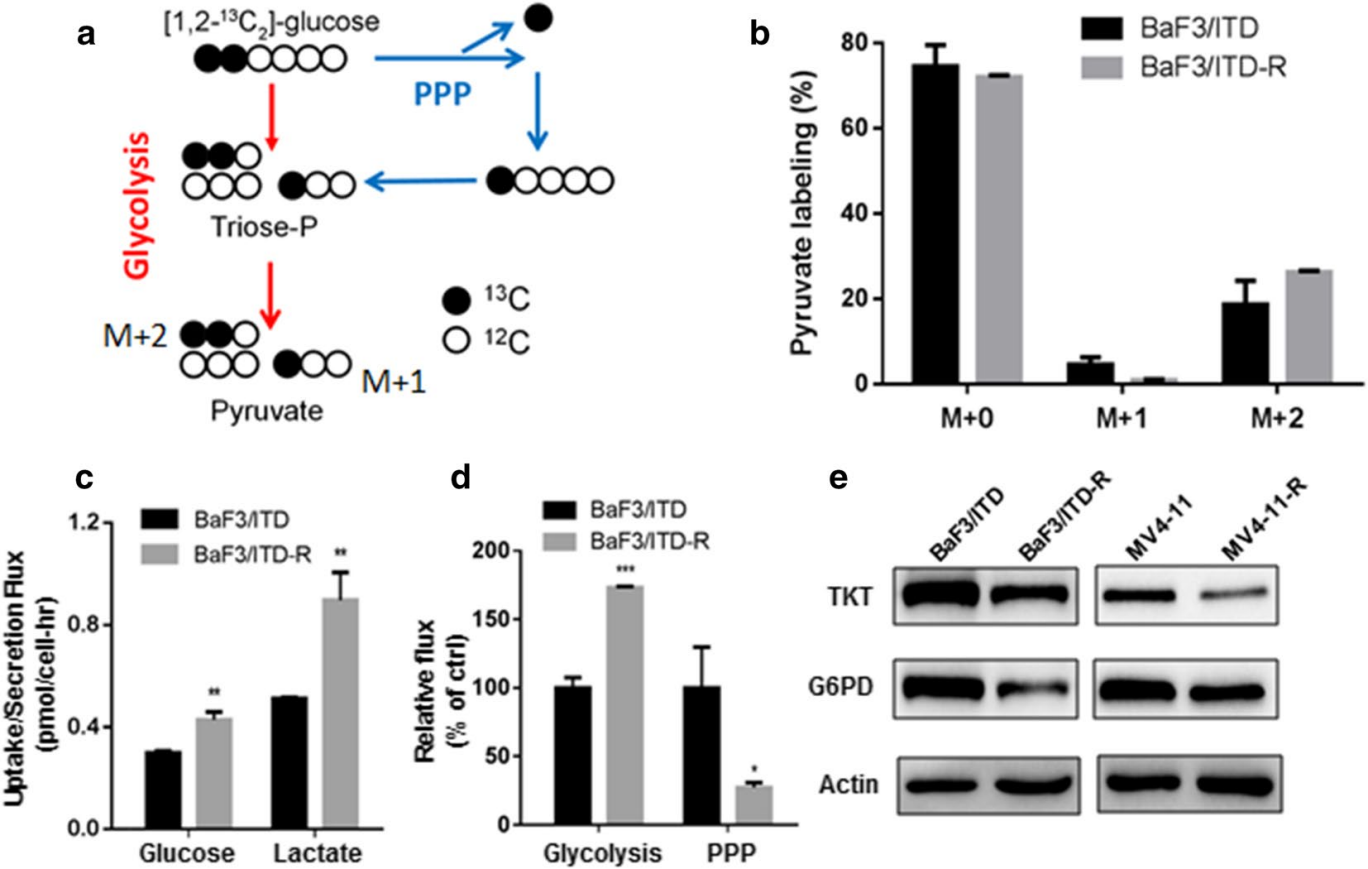
Illustration of the decreased glucose flux into the pentose phosphate pathway (PPP) in BaF3/ITD-R cells. **a** Schematic of [1,2-^13^C_2_]-glucose metabolism. The labeled glucose is converted to M + 1 pyruvate through the PPP and M + 2 pyruvate through glycolysis, respectively. **b** Quantification of mass isotopomer distribution of pyruvate in BaF3/ITD and BaF3/ITD-R cells cultured in [1,2-^13^C_2_]-glucose for 24 h. **c** Glucose uptake and lactate secretion in BaF3/ITD and BaF3/ITD-R cells as described in “Materials and methods” section . **d** The glucose flux through glycolysis and the PPP in BaF3/ITD and BaF3/ITD-R cells was calculated as described in “Materials and methods” section. **e** Western blot analysis of TKT and G6PD in BaF3, BaF3/ITD-R, MV4-11 and MV4-11-R cells. Bars, mean ± SEM (Standard Error of the Mean). *P < 0.05, **P < 0.01, ***P < 0.001. *PPP* pentose phosphate pathway; *TKT* transketolase; *G6PD* glucose-6-phosphate dehydrogenase

As ribose-1-phosphate could be further converted to ribose-5-phosphate, which is an intermediate of the non-oxidative phase of the PPP and PPP recycles intermediates back to the glycolytic pathway, we cultured the cells in the presence of [1,2-^13^C_2_]-glucose to quantify the amount of labeled and unlabeled pyruvate by GC–MS, to measure the direction of the metabolic flux related to the resistance to sorafenib. As shown on Fig. 3a, [1-^13^C_1_]-pyruvate (M + 1) indicates the amount of glucose entering the PPP and recycling back to glycolysis, and [1,2-^13^C_2_]-pyruvate (M + 2) indicates the amount of glucose converted to pyruvate in the glycolytic pathway. Figure 3b shows the amount of unlabeled pyruvate (M + 0) and isotopologues of pyruvate (M + 1, M + 2) in BaF3/ITD and BaF3/ITD-R cells. It is known that glycolysis is a metabolic pathway that converts glucose to pyruvate, which is then reduced by lactate dehydrogenase to produce lactate, resulting in a net production and extrusion of protons into the extracellular medium. Based on analysis of extracellular fluxes, we observed significant increases in glucose uptake and lactate production in the resistant cells (Fig. 3c). The ratio of M + 1/(M + 1) + (M + 2) multiplied by extracellular glucose uptake indicates the metabolic flux of glucose into the PPP. As shown in Fig. 3d, the resistant cells exhibited an approximate 70% increase in glycolytic flux and a 70% decrease in PPP flux, as compared with the sensitive cells. We further used real-time PCR and western blot analysis to evaluate the expression of enzymes in the PPP. As shown in Fig. 3e and Additional file 1: Figure S2, the majority of the enzymes including G6PD in the oxidative arm and TKT in the non-oxidative arm were downregulated in both BaF3/ITD-R and MV4-11-R cells when compared with the expression levels in their sensitive counterparts. In addition, we observed a consistent increase of intermediates in the glycolytic pathway and decrease of intermediates in the tricarboxylic acid (TCA) cycle in the BaF3/ITD-R cells compared with the parental cells (Additional file 1: Figure S5).

### Rewired glucose flux in BaF3/ITD-R cells is associated with reduced NADPH level and cell proliferation

**Fig. 4:**
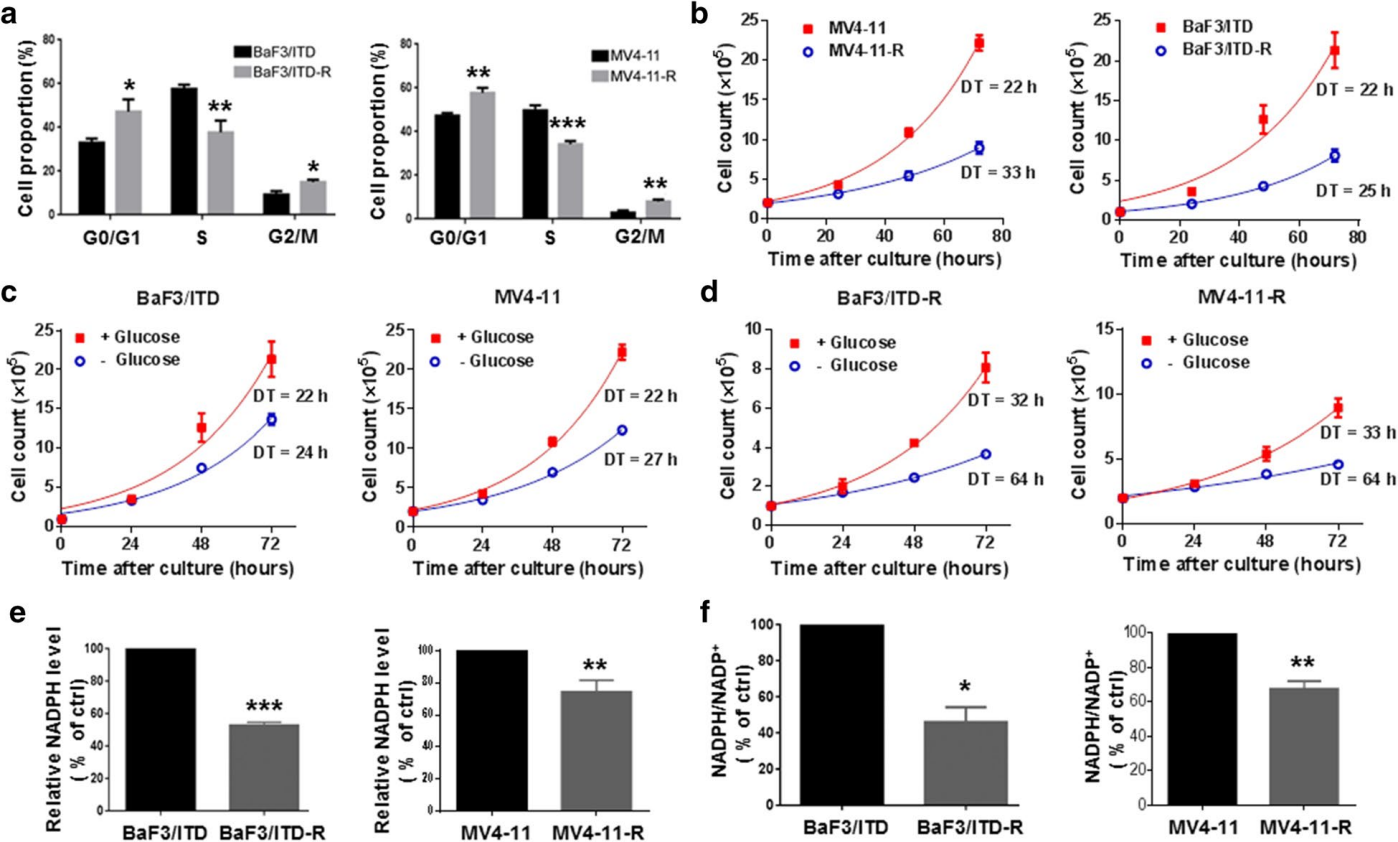
The sorafenib-resistant cells display reduced proliferation, higher demand for glucose, and decreased NADPH levels. **a** Cycle distribution analysis of BaF3/ITD, BaF3/ITD-R, MV4-11 and MV4-11-R cells. **b** Proliferation curves determined by the direct counting of the BaF3/ITD, BaF3/ITD-R, MV4-11 and MV4-11-R cells for 72 h. **P < 0.01, ***P < 0.001. **c** Proliferation curves of BaF3/ITD and MV4-11 cells with or without glucose in the culture medium. **P < 0.01, ***P < 0.001, vs. cells cultured with glucose in the medium. **d** Proliferation curves of BaF3/ITD-R and MV4-11-R cells with or without glucose in the culture medium. **e**,**f** Comparison of NADPH level and NADPH/NADP^+^ ratio in sensitive and resistant cells. Bars, mean ± SEM. **P < 0.01, ***P < 0.001, vs. sensitive cells. *DT* doubling time, *NADPH* nicotinamide adenine dinucleotide phosphate

Given that the PPP produces ribose 5-phosphate (R5P) and provides precursors for nucleotide synthesis, we compared the cell proliferation of the sensitive and resistant cells. The results of cell cycle analysis (Fig. 4a) showed a significant decrease in the population of resistant cells in the S phase compared to that of sensitive cells. Consistently, the resistant cells showed a decreased rate of proliferation in comparison with the sensitive cells (Fig. 4b). The cell proliferation assay by direct cell counting also indicated that the sensitive BaF3/ITD and MV4-11 cells exhibited similar doubling time when cultured in normal medium and glucose-free medium (22 h vs. 24 h for BaF3/ITD and 22 h vs. 27 h for MV4-11) (Fig. 4c). In contrast, the doubling time was significantly increased for resistant cells cultured in glucose-free medium compared with those cultured in normal medium (59 h vs. 35 h for BaF3/ITD-R, and 64 h vs. 33 h for MV4-11-R) (Fig. 4d). Our results suggest that reduced glucose flux through the PPP may affect cell growth and, hence, the resistant cells are highly dependent on glucose for cell proliferation. It is known that the oxidative arm of the PPP also provides NADPH for the regeneration of glutathione (GSH) to maintain a redox balance. We found that NADPH levels and the ratio of NADPH/NADP^+^ were both decreased in the resistant cells (Fig. 4e, f).

### Sorafenib-resistant cells exhibit an increase in antioxidant capacity

**Fig. 5:**
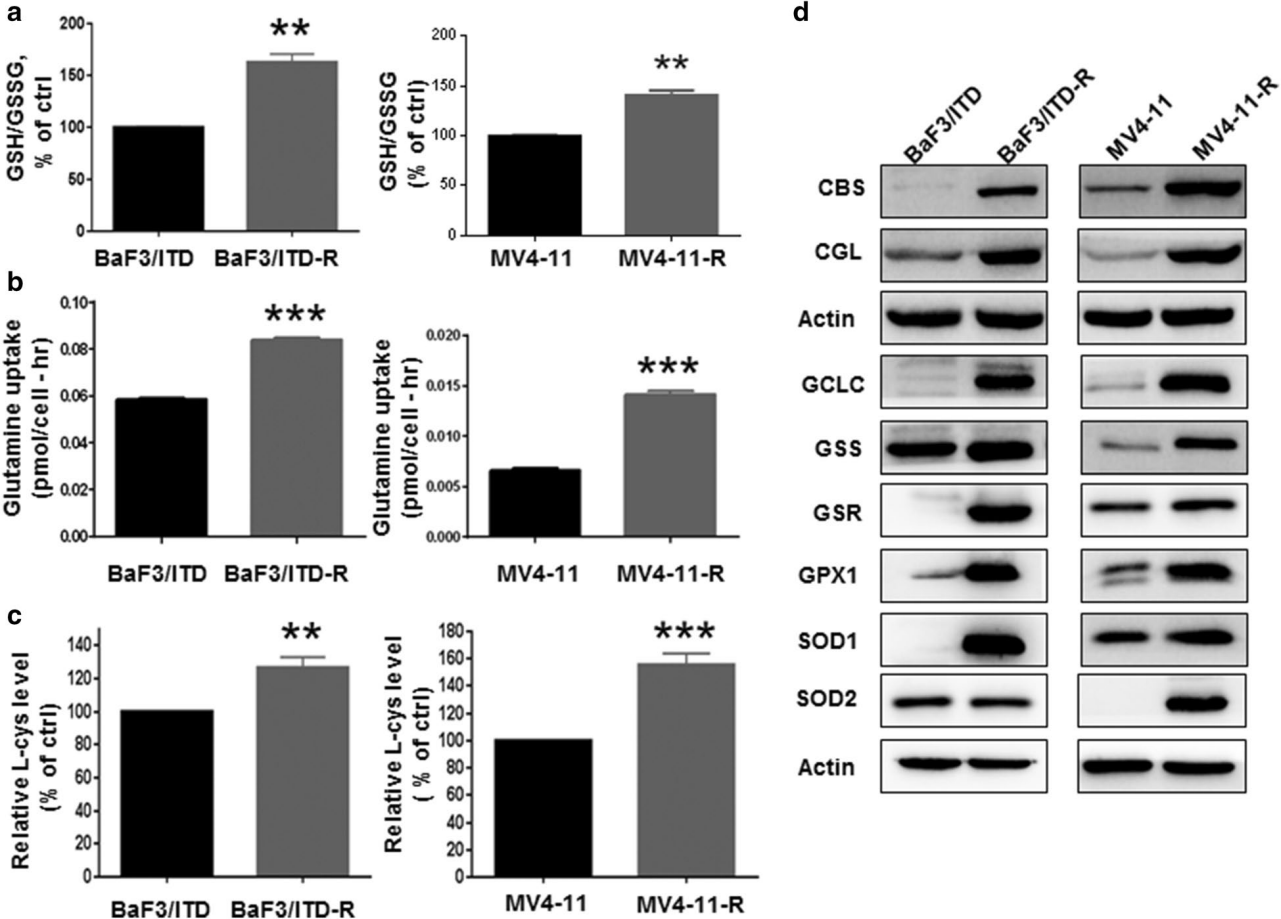
Enhanced antioxidant capacity in sorafenib-resistant cells. Comparison of the **a** GSH/GSSG ratio, **b** glutamine uptake levels and **c** intracellular l-cysteine levels in the sensitive (BaF3/ITD and MV4-11) and resistant (BaF3/ITD-R and MV4-11-R) cells. **d** Immunoblotting analysis of major antioxidant enzymes and enzymes in the transsulfuration pathway in BaF3/ITD, BaF3/ITD-R, MV4-11, and MV4-11-R cells. Results are representative of three independent experiments. Bars, means ± SEM. **P < 0.01, ***P < 0.001, vs. sensitive cells. *GSH* glutathione, *GSSG* glutathione disulfide

As the PPP is a major biochemical pathway that generates NADPH to maintain the major redox molecule glutathione, we further assessed the glutathione level in the sensitive and resistant cells. Interestingly, both GSH level (Additional file 1: Figure S3) and the ratio of GSH/GSSG (Fig. 5a) were higher in the resistant cells than in the sensitive cells. We hypothesize that the upregulation of GSH level may be due to elevated biosynthesis of GSH from the precursors. Glutamate is an important precursor for GSH synthesis and is synthesized from glutamine by the enzyme glutaminase. We observed a significant increase in glutamine uptake from the cell culture medium of both BaF3/ITD-R and MV4-11 R cells, compared with its uptake by sensitive cells (Fig. 5b). Intracellular glutamate was indeed increased in the resistant cells, as measured by LC–MS (l-Glutamate, BaF3/ITD-R/ITD = 2.29-fold increase) (Table 1). Cysteine is the rate-limiting precursor for glutathione biosynthesis. We showed that BaF3/ITD-R and MV4-11-R cells exhibited approximately 25% and 50% increases in intracellular cysteine, respectively, when compared with the levels in the sensitive cells (Fig. 5c).

The transsulfuration pathway enzymes involving the interconversion of cysteine and homocysteine were also analyzed. As shown in Fig. 5d, both cystathionine-β-synthase (CBS) and Cystathionine-gamma-ligase (CGL) were substantially increased in both the BaF3/ITD-R and MV4-11-R cells. In addition, various glutathione-related enzymes, including the glutamate-cysteine ligase catalytic subunit (GCLC), glutathione synthetase (GSS), glutathione-disulfide reductase (GSR), and glutathione peroxidase (GPX1) were tested. Figure 5d demonstrates that the majority of enzymes involved in the glutathione synthesis and regeneration, including GCLC, GSS and GSR, were highly expressed in both the BaF3/ITD-R and MV4-11-R cells. Moreover, GPX1, the enzyme responsible for scavenging hydrogen peroxide (H_2_O_2_), was upregulated in the resistant cells, whereas no significant change in mitochondrial superoxide dismutase (SOD2) expression was detected, and the cytosolic form of superoxide dismutase (SOD1) was substantially increased in BaF3/ITD-R cells. Notably, both SOD1 and SOD2 were upregulated in MV4-11-R cells as compared with in the MV4-11 cells. Nuclear factor (erythroid-derived 2)-like 2, also known as Nrf2, is a master transcription factor that regulates the expression of a number of antioxidants including GCLC, GPX and SOD [14]. We also observed a marked increase in Nrf2 expression in both resistant cell lines (Additional file 1: Figure S4). Our results indicate that the sorafenib-resistant cells may be highly dependent on antioxidant defense system to counteract the potential harmful effect of ROS and maintain redox balance in a dynamic state.

## Discussion

Our study is the first to evaluate the resistance of FLT3/ITD-mutated leukemia cells to a tyrosine kinase inhibitor based on metabolomic study. Based on the metabolomic data, we observed that the sensitive BaF3/ITD and the resistant BaF3/ITD-R cells demonstrated different patterns of metabolism. IPA revealed that the top five altered metabolic networks in the resistant cells were related to purine metabolism, including the degradation and salvage pathways. Consistently, the most significantly altered (higher concentration in BaF3/ITD-R) metabolite was guanine, which belongs to the top ranked “purine ribonucleoside degradation to ribose-1 phosphate” pathway. It is known that ribose phosphate is an intermediate in the PPP and a precursor for purine synthesis. While glycolysis is the general route for glucose catabolism and energy formation in the cell, the PPP branches from glycolysis and plays a vital role by providing ribose phosphate for nucleotide synthesis and NADPH for fatty acid synthesis and redox metabolism [15]. Therefore, determination of the carbon flux distribution entering the oxidative branch of the PPP provides valuable insight into the functioning of a cell [16, 17] in addition to the global untargeted metabolomics. In the current study, we used heavy isotope-labeled glucose ([1,2-^13^C_2_]-glucose) in combination with glucose uptake and lactate secretion to determine the relative activities of both pathways. We demonstrated that the contribution of glucose carbon to the glycolytic pathway was increased while the flux of glucose through the PPP was decreased in the sorafenib-resistant cells.

The results of metabolic flux analysis observed in this study were in line with our previous observations on enhanced glycolytic enzyme expression and activity in the sorafenib-resistant cells [9]. Depletion of glucose and glycolytic inhibitors could indeed reverse such resistance. Consistent with this, 2-deoxy-d-glucose (2-DG) and 3-Bromopyruvate propyl ester (3-BrOP), inhibitors of the glycolytic enzyme hexokinase, were found to synergize with sorafenib in killing leukemia cells with FLT3/ITD mutation [18]. In addition, the increase of intermediates in the glycolytic pathway and decrease of intermediates in the TCA cycle in the BaF3/ITD-R cells, (Additional file 1: Figure S5), was also consistent with our recent study that demonstrated mitochondrial dysfunction in the sorafenib-resistant cells [9].

Futher, the majority of the enzymes were decreased in both oxidative arm and non-oxidative arm in the resistant cells, which signify that PPP is likely inhibited in the resistant cells. As most cancer cells depend on the de novo nucleotide synthesis for growth and survival [19], inhibition of the PPP appeared to associate with a reduced ability of cells to proliferate, as we observed a significant inhibition of cell growth in the sorafenib-resistant cells. Interestingly, RBKS (Ribokinase), an enzyme that catalyzes the phosphorylation of ribose appeared to be upregulated in the resistant cells. As the resulting D-ribose-5-phosphate can then be used for the synthesis of nucleotides, the upregulation of RBKS may be compensatory to the inhibition of PPP. The alteration of metabolic output from glucose to PPP in the resistant cells is understudied. Here, we found that glucose-derived carbons to PPP flux was reduced in the resistant cells. To better quantify how the oxidative and non-oxidative PPP flux contribute to ribose synthesis, measurement of the transfer of glucose carbons into RNA may be further required, as previously described by Zhao et al. [20]. We also observed a decrease in G6PD and TKT expression and NADPH levels in the sorafenib-resistant cells, compared with in their sensitive counterparts, although we did not provide direct evidence to show whether the decrease of NADPH is a result of decreased production or increased consumption of NADPH. A more direct approach to address the above question would be to use [^2^H]-glucose to trace NADPH production from the PPP and consumption by other metabolic pathways [12, 21].

**Fig. 6:**
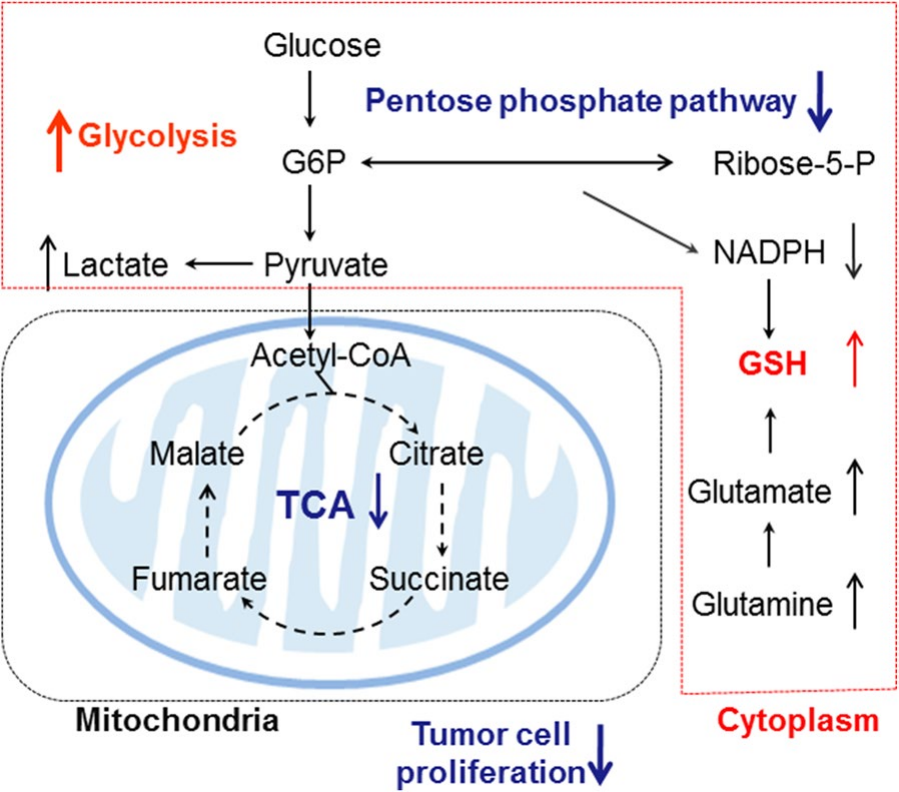
Metabolic rewiring of FLT3/ITD-mutated cells resistant to sorafenib. The resistant cells exhibit an increase in glycolytic activity, accompanied by a perturbed TCA cycle and inhibition of the pentose phosphate pathway, which may contribute to reduced proliferation of the resistant cells. GSH biosynthesis is increased in order to counteract the oxidative stress and to maintain a redox balance in a dynamic state. *G6P* glucose-6-phosphate, *R5P* ribose-5-phosphate, *TCA* tricarboxylic acid, *GSH* glutathione

NADPH is a major antioxidant required for the regeneration of reduced GSH to maintain redox homeostasis. The balance between ROS generation and elimination determines the cellular redox state. In the current study, most of the metabolites centralized to the glutathione production pathway were altered in the resistant cells. Methionine, through a series of reactions, gives rise to cysteine, which is the limiting factor for GSH synthesis [22]. Glutamate and glycine then enter the cysteine pathway to produce GSH. Our results showed that in the methionine-GSH pathway, the levels of methionine, cysteine, glutamate, and its precursor glutamine, were elevated in the BaF3/ITD-R cells compared with the parental cells. It is known that the turnover of GSSG to GSH requires NADPH. However, in the current study, we observed that the sorafenib-resistant cells maintain a high GSH level under the low level of NADPH. We therefore hypothesize that the upregulation of glutathione reductase (GSR), which actively uses NADPH to reduce GSSG to GSH, may contribute to the decrease in NADPH and the relatively high GSH in the drug-resistant cells. Overall, the resistant cells presented high levels of both GSH and GSH-related enzymes including GCLC, GSS, GSR and GPX. Thus, the metabolomic analysis, along with the expression data on the enzymes catalyzing the reactions in the pathway, integrates the levels of input, intermediates and outcome, and provides a comprehensive understanding of the functional significance of the GSH pathway in the sorafenib-resistant cells. Our data suggest that the upregulation of antioxidant capacity in response to intrinsic oxidative stress may be associated with the development of sorafenib resistance. It should be noted that Nrf2 plays an important role in regulating a number of major antioxidants including GCLC, GPX, GST, and SOD, [14]. Nrf2 was also found to be upregulated in the sorafenib-resistant cells. Future studies shall be conducted to further define the role of Nrf2 and the downstream antioxidants in resistance to sorafenib. Moreover, while lower enzyme expression or activity of G6PD in the PPP exists in drug-resistant cells [23, 24], other studies have also found that an active PPP with elevated levels of NADPH may contribute to multidrug resistance in experimental models [25–27]. Given the heterogeneous nature of redox and metabolic status, characterizing the reciprocal interplay between metabolic pathways may provide an improved understanding of tumor progression and drug resistance (Fig. 6).

There are several limitations to this study that should be addressed. First, although we observed a decreased activity of PPP, however due to technical limitation, we were unable to differentiate the contribution of the oxidative and the non-oxidative pathway. Second, the alteration of metabolomic profile of sorafenib-resistant cells was only tested in the BaF3/ITD-R cells and needs to be confirmed in other model systems. Third, the inhibition of glycolysis and GSH shall be further tested in vivo to investigate alternative therapeutic agents for leukemia resistant to FLT3 inhibitors.

## Conclusions

In summary, we performed a metabolomic study and illustrated the metabolic shifts metabolic and redox adaptation that may be associated with drug resistance in FLT3/ITD-mutated cells that can tolerate the chronic exposure to the FLT3 tyrosine kinase inhibitor sorafenib. The key metabolic alterations in the resistant cells were identified to enhance glycolytic activity, accompanied by a perturbed TCA cycle and a reduced rate of PPP flux, and an increased antioxidant capacity of GSH.

## Additional file

**Additional file 1: Figure S1.** Illustration of the ingenuity pathway analysis (IPA). **Figure S2.** Real-time PCR analysis of PPP enzymes in the sensitive and resistant cells. **Figure S3.** GSH levels in the sensitive and resistant cells. **Figure S4.** Western blot analysis of Nrf2 in the sensitive and resistant cells. **Figure S5.** Relative intracellular metabolite abundance of BaF3/ITD and BaF3/ITD-R cells.

## Abbreviations

AML: acute myeloid leukemia
ITD: internal tandem duplications
FLT3: FMS-like tyrosine kinase-3
IPA: ingenuity pathway analysis
PI3 K: phosphatidylinositide 3-kinase
AKT: protein kinase B
MAPK: mitogen-activated protein kinase
ERK: extracellular-regulated protein kinases
TKT: transketolase
PPP: pentose phosphate pathway
G6P: glucose-6-phosphate
G6PD: glucose-6-phosphate dehydrogenase
R5P: ribose-5-phosphate
NADPH: nicotinamide adenine dinucleotide phosphate
ROS: reactive oxygen species
TCA: tricarboxylic acid
MDR: multidrug resistance
GSH: glutathione
GSSG: glutathione disulfide
CBS: cystathionine-β-synthase
CGL: cystathionine-gamma-ligase
GCLC: glutamate-cysteine ligase catalytic subunit
GSS: glutathione synthetase
GSR: glutathione-disulfide reductase
GPX1: glutathione peroxidase 1
SOD1: superoxide dismutase 1
SOD2: superoxide dismutase 2
Nrf2: nuclear factor E2-related factor 2
