# Supplementary material for: Metabolic reprogramming and redox adaptation in sorafenib-resistant leukemia cells: detected by untargeted metabolomics and stable isotope tracing analysis
Source: Cancer Commun (Lond). 2019 Apr 4;39:17. doi: 10.1186/s40880-019-0362-z (PMC6449955; doi:10.1186/s40880-019-0362-z)
Supplement: Supplementary file 1 — Additional file 1: Figure S1. Illustration of the ingenuity pathway analysis (IPA). Figure S2. Real-time PCR analysis of PPP enzymes in the sensitive and resistant cells. Figure S3. GSH levels in the sensitive and resistant cells. Figure S4. Western blot analysis of Nrf2 in the sensitive and resistant cells. Figure S5. Relative intracellular metabolite abundance of BaF3/ITD and BaF3/ITD-R cells. [file 40880_2019_362_MOESM1_ESM.docx]

**Additional Figure S1**

**
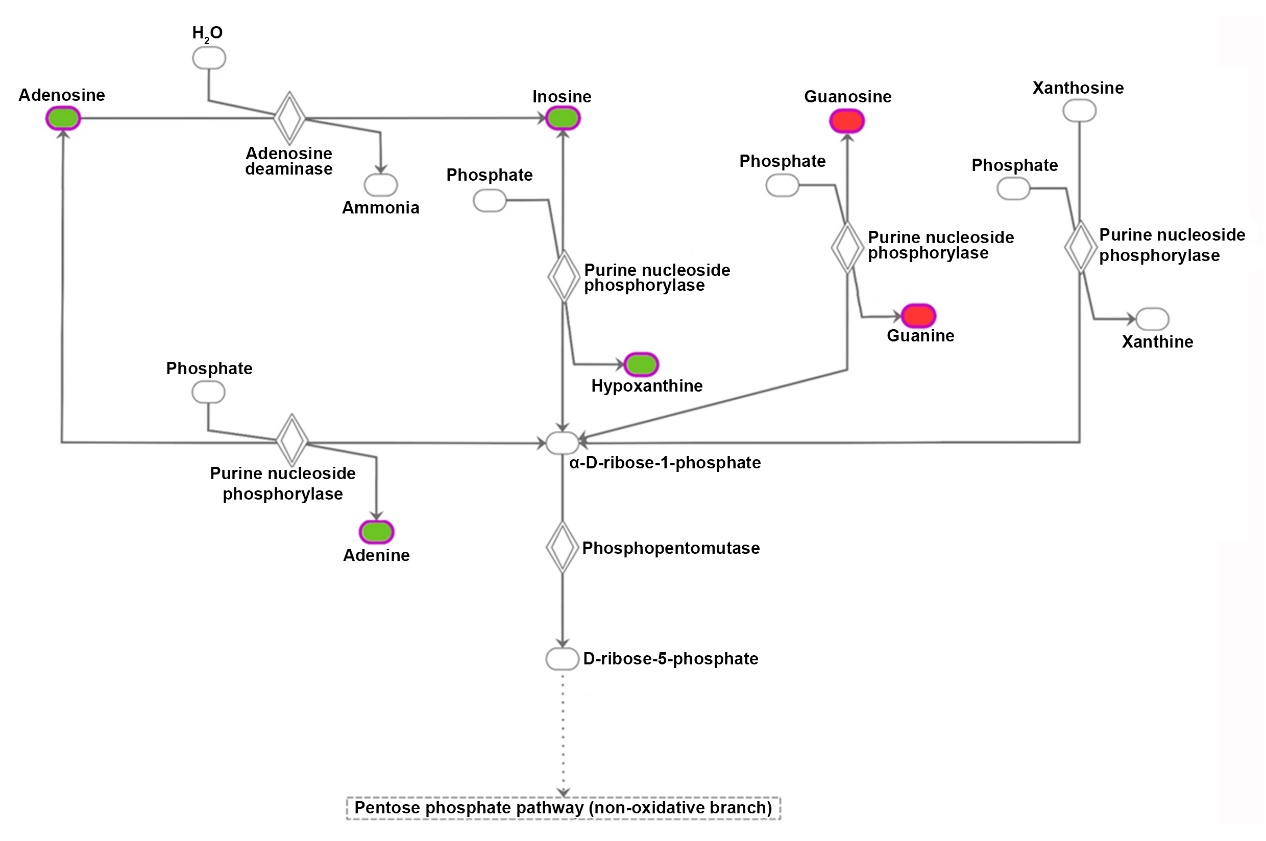
**

**Additional Figure S1. Illustration of the ingenuity pathway analysis (IPA).** The pentose phosphate pathway is shown as the highest ranked canonical pathway with significant changes in BaF3/ITD-R cells, as analyzed by the IPA. Molecules with red marker represent metabolites increased in BaF3/ITD-R. Molecules with green marker represent metabolites decreased in BaF3/ITD-R. Molecules with white marker represent metabolites with no change in BaF3/ITD-R cells.

**Additional Figure S2**

**
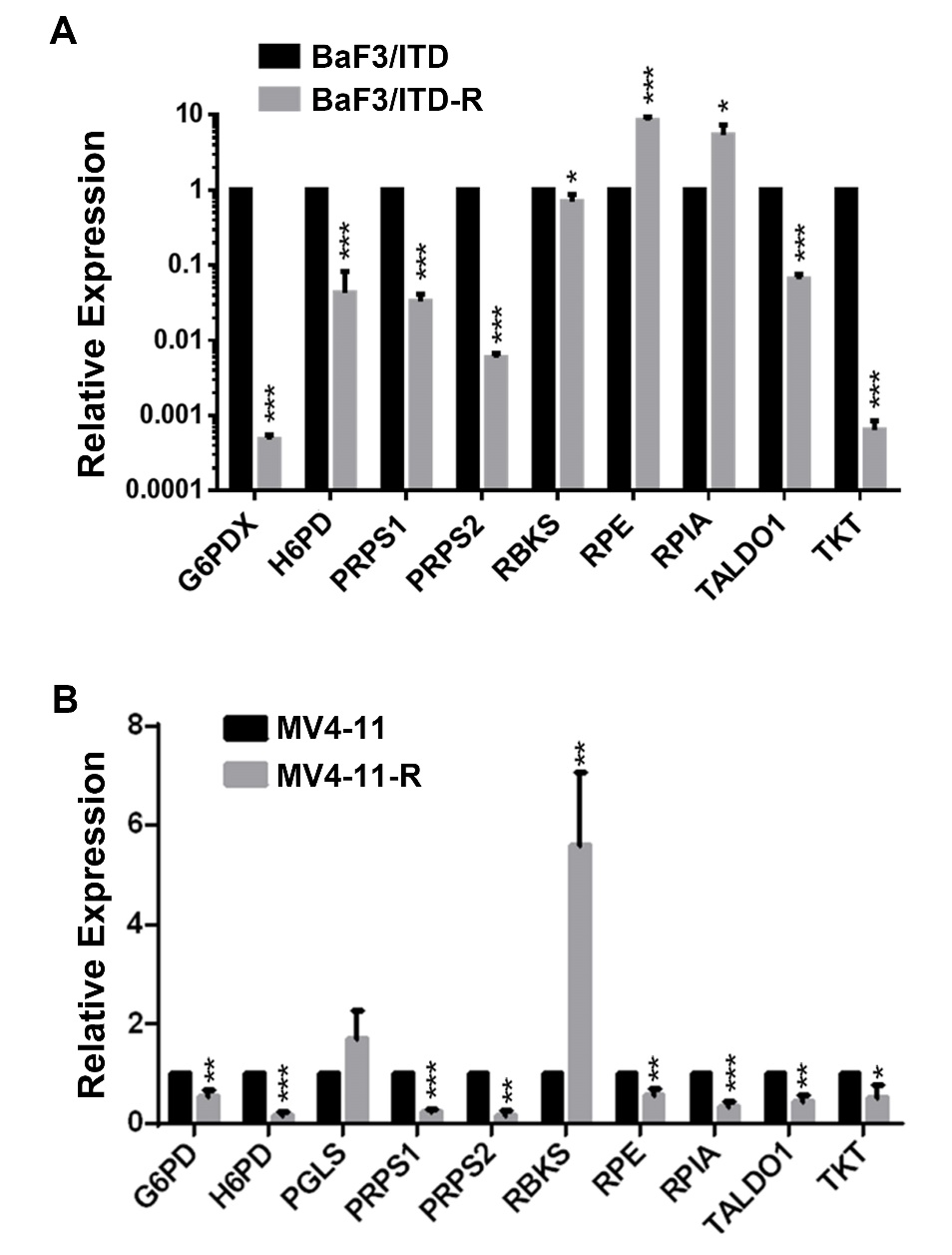
**

**Additional Figure S2. Real-time PCR analysis of PPP enzymes in the sensitive and resistant cells.** BaF3/ITD vs. BaF3/ITD-R (A) and MV4-11 vs. MV4-11-R (B): Bars, means ± SEM. * *P* < 0.05, ** *P* < 0.01, *** *P* < 0.001.

Abbreviations: PPP, pentose phosphate pathway; G6PDX, Glucose-6-phosphate 1-dehydrogenase X; H6PD, GDH/6PGL endoplasmic bifunctional protein; PRPS1, Ribose-phosphate pyrophosphokinase 1; PRPS2, Ribose-phosphate pyrophosphokinase 2; RBKS, Ribokinase; RPE，**Ribulose-phosphate 3-epimerase; RPIA, Ribose-5-phosphate isomerase; TALDO1, Transaldolase；TKT, Transketolase;** G6PD, Glucose-6-phosphate dehydrogenase; PGLS, **6-phosphogluconolactonase**

**Additional Figure S3**


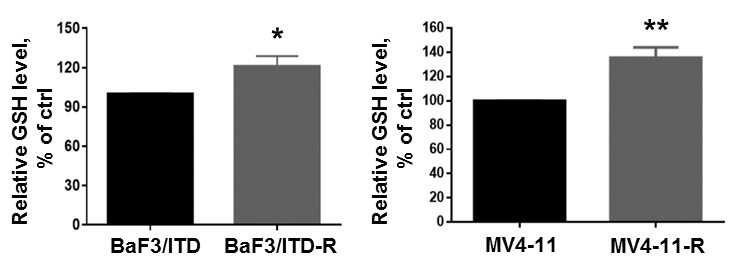


**Additional Figure S3. GSH levels in the sensitive and resistant cells.** BaF3/ITD vs. BaF3/ITD-R and MV4-11 vs. MV4-11-R. * *P* < 0.05, ** *P* < 0.01. Abbreviations: ctrl, control; GSH, glutathione

**Additional Figure S4**

**
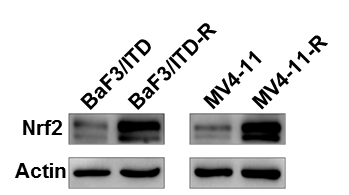
**

**Additional Figure S4. Western blot analysis of Nrf2 in the sensitive and resistant cells**. BaF3/ITD vs. BaF3/ITD-R and MV4-11 vs. MV4-11-R.

**Additional Figure S5**

**
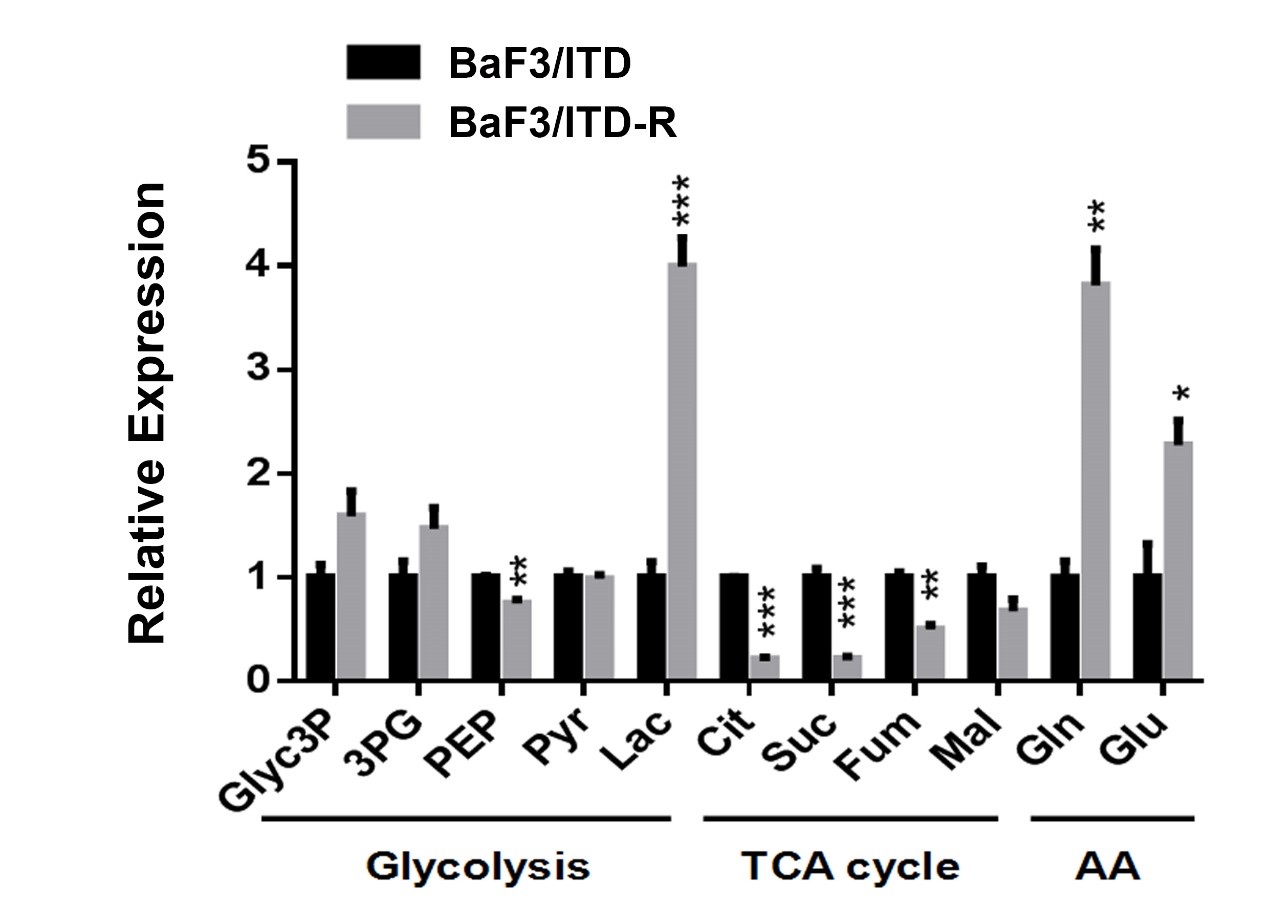
**

**Additional Figure S5. Relative intracellular metabolite abundance of BaF3/ITD and BaF3/ITD-R cells**. Metabolite extraction and GC-MS analysis was described in section “Method and materials”.

Abbreviation: Glyc3P, glyceraldehydes 3-phosphate; 3PG, 3-Phosphoglyceric acid; PEP, Phosphoenolpyruvate; Pyr, pyruvate; Lac, lactate; Cit, citrate; Suc, succinate; Fum, fumarate; Mal, malate; Gln, glutamine; Glu, glutamate; TCA cycle, tricarboxylic acid; AA, amino acid.
